# Nutritional Quality of Meat Analogues: Results From the Food Labelling of Italian Products (FLIP) Project

**DOI:** 10.3389/fnut.2022.852831

**Published:** 2022-04-26

**Authors:** Sara Cutroneo, Donato Angelino, Tullia Tedeschi, Nicoletta Pellegrini, Daniela Martini, SINU Young Working Group, Margherita Dall'Asta

**Affiliations:** ^1^Food and Drug Department, University of Parma, Parma, Italy; ^2^Faculty of Bioscience and Agro-Food and Environmental Technology, University of Teramo, Teramo, Italy; ^3^Department of Agricultural, Food, Environmental and Animal Sciences, University of Udine, Udine, Italy; ^4^Department of Food, Environmental and Nutritional Sciences, University of Milan, Milan, Italy

**Keywords:** meat analogues, food labelling, food quality, plant-based meat substitutes, Nutri-Score

## Abstract

Nowadays, the interest in meat substitutes is increasing, and consumers perceive their nutritional quality better than that of the animal products they intend to resemble. Therefore, this work aimed to investigate the overall nutritional quality of these new products. Regulated information [Regulation (EU) 1169/2011], the presence/absence of nutrition or health claim and organic declarations, the gluten-free indication, and the number of ingredients were collected from the food labels of 269 commercial meat analogues currently sold on the Italian market. Nutritional information of reference animal meat products was used to compare the nutrition profile. As an indicator of the nutritional quality, the Nutri-Score of meat analogues and counterparts was also determined. Plant-based steaks showed significantly higher protein, lower energy, fats and salt contents, and better Nutri-Scores than the other analogues. All the meat analogues showed a higher fibre content than meat products, while plant-based burgers and meatballs had lower protein contents than meat counterparts. Ready-sliced meat analogues showed a lower salt content than cured meats. Overall, all these plant-based products showed a longer list of ingredients than animal meat products. Results from this survey highlighted that plant-based steaks, cutlets, and cured meats have some favourable nutritional aspects compared to animal-based products. However, they cannot be considered a “*tout-court*” alternative to meat products from a nutritional point of view.

## Introduction

The high consumption of red meat and mostly processed meat is currently debated for many reasons related to human, planet, and animal health. First, diets high in red meat and processed meat have been considered risk factors for a high number of deaths and years lived with disability (DALYs) in the last Global Burden of Disease study (1). However, other dietary risk factors, such as low consumption of whole grains, fruit, and vegetables or high sodium intake, have been attributed to an even higher number of deaths and DALYs (1). In particular, processed meat has been classified as carcinogenic to humans by the International Agency for Research on Cancer, mainly for the relationship between its high consumption and the incidence of colorectal cancer (2). Secondly, meat and animal foods are generally associated with a higher environmental impact in terms of global greenhouse gasses (GHG), land, and water use compared to plant foods (3). This represents an urgent issue to be considered since food production emits one-third of total GHGs, occupies ~40% of Earth's land and uses over two-thirds of Earth's freshwater (4).

For all these reasons, there is currently an urgent call to promote plant-based diets limiting the consumption of meat and animal foods. Plant-based diets foresee the inclusion of crops (e.g., cereals, legumes) and their traditional derivatives (e.g., pasta), but also of a large plethora of plant-based foods developed to mimic animal foods. These products include plant-based drinks, cheese substitutes, and even meat analogues, with the latter reaching a 4.6 USD billion global market in 2020 and expecting 6 USD billion within 4 years (5). It is also expected that 39% of the revenue will be concentrated in Europe (5).

Meat analogues—also known as meat substitutes—are plant-derived food products usually processed to resemble meat flavour, texture, and appearance. They can be derived from various vegetable sources. Soy and textured vegetable proteins are the most common, but pulses (i.e., peas, lentils, beans, and lupin), cereals (i.e., wheat, quinoa, amaranth, and buckwheat), mushrooms, and seeds (i.e., linseeds) are also used (5, 6). More, these products are added with several other ingredients, such as protein and polysaccharides, which are the building blocks of the product to mimic the meat structure, and vegetable oils and spices to contribute to both texture and flavour. Pigments are generally added to affect the product colour, while vitamins and minerals, and preservatives are often added to improve nutritional quality and shelf-life, respectively (7).

Meat analogues can be produced with two different approaches aiming at obtaining a fibrous structure, which is essential for the texture (8). In the first one, the ingredients are structured and then assembled to form the final product (i.e., cultured meats, mycoprotein-based products). In contrast, in the second one, the formation of a fibrous structure starts from a mix of proteins and polysaccharides is used. Depending on the final product to be obtained, different combinations of processes and ingredients are used so that each analogue has different physical, nutritional, and sensory characteristics (9).

The interest in these innovative products and, consequently, their purchase in supermarkets is constantly growing. Moreover, these products are perceived by consumers as healthier than meat (6), even though little is known about their real nutritional quality. In this regard, the purposes of this work were to (i) investigate the nutritional composition of the commercial plant-based meat analogues sold in Italy by collecting the nutrition facts on their packaging; (ii) perform a comparison, in terms of nutritional values, of these products with the meat ones they intend to resemble; (iii) determine the Nutri-Score of these new products and the animal-based products as an indicator of nutritional quality.

This work is part of the Food Labelling of Italian Products (FLIP) study that systematically investigates the overall quality of the pre-packed foods of the most important food groups and related categories sold on the Italian market.

## Materials and Methods

### Sample Selection

The selection of samples was performed, as previously described by Angelino et al. (10), in the online stores of the major retailers currently present on the Italian market (11): Bennet drive, Carrefour, Conad, Coop, Crai, Despar, Esselunga, Il gigante, Iper, Panorama, Selex. Since these products are often sold in specialised shops in organic products, two other retailers (Macrolibrarsi, NaturaSì) were included to have a more representative sample selection. The search was performed between April 2020 and August 2020.

The inclusion criteria taken into consideration for the selection were the item's availability in at least one online shop and all the data to be retrieved on the pack or in the retailer's online shop. Exclusion criteria were: incomplete images of all the sides of the pack, unclear images of nutrition declaration or list of ingredients, and products marked as “product currently unavailable” on all the selected online stores during the data collection period.

### Control Selection

The comparison of the nutritional values of meat-alternatives products with meat product counterparts was performed by considering two types of control products. For steak and cured meats, nutritional values were retrieved from the Food Composition Database for Epidemiological Studies (12). For cured meats, except for those undergoing a cooking treatment (e.g., stir-fry sausage), all items were retrieved. For steak controls, the selected products were: horse and donkey; bovine/steer/veal; calf; poultry; pork; rabbit and hare meat; *Caprinae* meat (sheep, lamb, goat); other animals (12). The inclusion criteria were the type of meat and meat cuts traditionally used for a steak. Therefore, offal and non-specific cuts for the meat as well as cooked products were excluded. Steak controls were divided into red meat (cattle, horse, pork) and white meat (chicken, turkey, rabbit, lamb, kid).

Commercial products were selected for all the other meat controls (burgers, meatballs, and cutlets) and the related energy and nutrient contents were retrieved as described for meat analogues.

### Data Extraction

Data extraction was conducted as described by Angelino et al. (13). For each product, regulated information [according to the Council Regulation (EU) 1169/2011 (14)] was collected: descriptive name, energy (kcal/100 g and kJ/100 g), total fat (g/100 g), saturates (g/100 g), carbohydrates (g/100 g), sugars (g/100 g), protein (g/100 g), salt (g/100 g), and the list of ingredients. Also, the presence/absence of nutrition claim and health claim declaration [according to the Council Regulation (EC) 1924/2006 (15)], and organic declaration [according to the Council Regulation (EC) No. 834/2007 (16)] were collected.

The following additional information was collected: fibre content (g/100 g), number of ingredients, presence/absence of a gluten-free indication, and plant they were made of (i.e., vegetable, legumes, cereals).

Collected data were organised in a dataset where commercial products were sub-grouped depending on: (1) category, (2) type, (3) presence/absence of an organic declaration, (4) presence/absence of nutrition claims, (5) presence/absence of health claims, (6) presence/absence of indications related to gluten. All the plant-based products investigated were divided into two major categories: plant-based meat analogues (PBMA) and plant-based ready-sliced meat analogues (PBSMA). Among the PBMA, we identified different types of products they intend to resemble, i.e., steaks, burgers, meatballs, and cutlets. As for the PBMA, we considered as control products: steaks, both white meat and red meat, burgers, meatballs, and cutlets.

### Determination of the Nutri-Score

For all the products, the Nutri-Score was also determined by using the Excel sheet provided by the *Santé publique*—France (17). Sodium (mg/100 g) was calculated by dividing the salt value reported in the nutrition declaration (g/100 g) per 2.5 and converted it to mg. For the samples without indication of fibre content (*n* = 15 PBMA, *n* = 5 PBSMA, and *n* = 153 controls), this value was estimated by subtracting the energy provided by each macronutrient from the total energy, divided by 2 kcal/g. The percentage of fruit, vegetables, pulses, nuts and rapeseed, walnut, and olive oil was retrieved from the list of ingredients. As the salt is added to steak during cooking, different amounts of salt (from 0.05 to 0.5 g) were considered to determine the score of this control product. In comparing the analogue products and the meat counterparts, 0.5 g of salt was considered. For meat products retrieved in the Food Composition Database for Epidemiological Studies, Nutri-score was calculated considering nutrition values reported in the database, even though this type of front-of-pack labelling is not applied to the not pre-packed products.

### Statistical Analysis

The statistical analysis was carried out using the Statistical Package for Social Sciences software (IBM SPSS Statistics, Version 26.0, IBM corp., Chicago, IL) and performed at *p* < 0.05 of the significance level. The normality of data distribution was rejected through the Kolmogorov–Smirnov test. Therefore, data related to energy, macronutrient, fibre, and salt were expressed as median and interquartile ranges. In order to investigate differences among categories, the Kruskal–Wallis non-parametric one-way ANOVA for independent samples with multiple pairwise comparisons test was used. Differences between products with or without organic declaration, nutrition and health claim declaration, and indication related to gluten were assessed by using the Mann–Whitney non-parametric test for two independent samples. In addition, nutritional values of meat replacer products were compared to those of control counterparts using the Mann–Whitney non-parametric test for two independent samples. Results were graphically shown using Origin software (OriginPro 2019, OriginLab corp., Northampton, MA).

## Results

Two hundred and ninety meat analogues were collected. After removing products not respecting inclusion criteria, a total of 269 products were considered in the final analysis, 229 PBMA and 40 PBSMA (Tables 1, 2). PBMA were subcategorized in steaks (29.7%), burgers (45.9%), meatballs (9.6%), and cutlets (14.8%). Definitions and examples of the categorisation of the meat analogues are reported in Supplementary Table 1.

**Table 1:** The number of items and nutrition declaration [according to the Council Regulation (EU) 1169/201 (14)] of plant-based meat analogues reported for each category, presence of nutrition claim, organic declaration, and gluten-free indication.

		**Number of items**	**Energy kcal/100 g**	**Total fat g/100 g**	**Saturates g/100 g**	**Total carbohydrates g/100 g**	**Sugars g/100 g**	**Fibre g/100 g**	**Protein g/100 g**	**Salt g/100 g**
All meat analogues		229	198 (155–230)	8.7 (5.8–11.9)	1.3 (0.9–1.6)	12.5 (5.0–18.0)	1.3 (0.5–2.6)	4.0 (2.6–5.7)	14.0 (9.8–17.0)	1.2 (0.9–1.5)
Category	Steaks	68	153 (135–179)^c^	6.5 (2.0–8.7)^b^	1.0 (0.4–1.4)^b^	3.4 (1.7–5.8)^c^	0.4 (0.1–0.8) ^b^	1.9 (1.2–3.5)^b^	17.6 (14.3–23.7)^a^	0.7 (0.1–1.6)^c^
	Burgers	105	209 (176–233)^b^	10.6 (7.0–13.0)^a^	1.3 (1.0–1.9)^a^	14.0 (10.0–17.8)^b^	2.0 (1.0–3.1)^a^	4.7 (3.9–6.5)^a^	12.0 (6.8–15.0)^b^	1.2 (0.9–1.5)^b^
	Meatballs	22	221 (178–255)^ab^	10.8 (6.0–14.6)^a^	1.5 (1.1–2.2)^a^	13.8 (8.7–23.8)^b^	2.3 (1.0–2.8)^a^	4.5 (3.6–6.8)^a^	12.6 (7.8–16.0)^b^	1.3 (1.1–1.6)^bc^
	Cutlets	34	228 (221–242)^a^	10.3 (8.0–11.9)^a^	1.3 (1.0–1.5)^a^	20.4 (17.5–24.0)^a^	1.7 (1.0–3.1)^a^	3.8 (3.4–5.0)^a^	12.8 (11.0–15.0)^b^	1.5 (1.1–1.8)^a^
Nutrition claim	No	63	196 (161–228)	10.0 (5.0–12.4)	1.4 (0.9–2.0)*	13.1 (3.3–19.0)	1.7 (0.5–2.5)	4.4 (2.3–7.0)	11.9 (6.6–16.4)*	1.2 (0.9–1.7)
	Yes	166	198 (154–231)	8.5 (6.1–11.7)	1.2 (0.9–1.5)	12.3 (5.3–17.5)	1.2 (0.5–2.7)	4.0 (2.7–5.5)	14.3 (11.8–17.3)	1.2 (0.8–1.5)
Fat claim	No	196	199 (156–232)	9.1 (6.5–12.2)*	1.3 (1.0–1.7)*	13.1 (4.9–18.1)	1.3 (0.4–2.4)*	4.0 (2.4–5.7)	13.9 (8.9–16.9)*	1.2 (0.8–1.5)
	Yes	33	184 (141–217)	7.5 (2.6–10.1)	0.9 (0.5–1.2)	10.0 (5.6–15.7)	1.8 (1.0–3.5)	4.1 (3.2–6.5)	16.0 (13.8–19.0)	1.4 (1.0–1.6)
Protein claim	No	74	195 (163–227)	9.8 (5.0–12.7)	1.5 (0.9–2.0)*	13.6 (3.5–20.0)	1.6 (0.5–2.4)	4.5 (2.4–6.9)	11.0 (6.5–16.4)*	1.3 (0.9–1.7)
	Yes	155	198 (154–232)	8.4 (6.1–11.0)	1.2 (0.9–1.5)	12.0 (5.0–17.0)	1.2 (0.5–2.7)	4.0 (2.7−5.2)	14.3 (12.0–17.3)	1.2 (0.8–1.5)
Fibre claim	No	150	184 (147–229)*	8.4 (4.6–11.7)	1.3 (0.9–1.6)	10.7 (3.4–18.0)*	1.0 (0.4–2.1)*	3.4 (1.8–5.0)*	14.1 (10.0–17.6)	1.1 (0.8–1.5)*
	Yes	79	211 (188–235)	9.3 (6.8–12.7)	1.3 (1.0–1.7)	14.2 (9.3–19.0)	1.9 (0.7–3.5)	4.8 (3.9–6.8)	13.9 (8.8–16.0)	1.3 (1.0–1.7)
Organic	No	102	215 (172–233)*	10.3 (7.0–12.7)*	1.3 (0.9–1.6)	13.5 (6.5–18.0)	1.5 (0.7–2.7)*	4.3 (3.4–6.3)*	13.9 (10.7–16.0)	1.3 (1.0–1.6)*
	Yes	127	187 (151–227)	7.8 (5.2–11.3)	1.3 (0.8–1.6)	11.0 (4.2–18.0)	1.1 (0.4–2.4)	3.8 (1.9–5.5)	14.1 (8.3–19.7)	1.1 (0.7–1.5)
Gluten free	No	198	203 (156–232)*	9.0 (5.7–12.3)	1.3 (0.9–1.7)*	12.6 (5.0–18.0)	1.3 (0.5–2.7)	4.0 (2.7–5.5)	14.0 (10.5–17.3)	1.3 (0.9–1.5)*
	Yes	31	180 (149–199)	7.9 (6.4–10.0)	1.0 (0.7–1.3)	10.6 (3.5–19.0)	1.5 (0.4–2.5)	5.0 (1.9–9.7)	13.8 (6.7–14.5)	0.9 (0.1–1.5)

*Data are expressed as median (25°–75° percentile). Different letters in the same column refer to significant differences among categories (Kruskal–Wallis non-parametric one-way ANOVA for independent samples with multiple pairwise comparisons test, p < 0.05). Asterisks within the same column indicate differences between items with or without nutrition claim declaration, organic declaration, and gluten-free indication (Mann–Whitney non-parametric test for two independent samples, p < 0.05)*.

**Table 2:** The number of items and nutrition declaration [according to the Council Regulation (EU) 1169/2011 (14)] of plant-based ready-sliced meat analogues reported for presence of nutrition claim and organic declaration.

		**Number of items**	**Energy kcal/100 g**	**Total fat g/100 g**	**Saturates g/100 g**	**Total carbohydrates g/100 g**	**Sugars g/100 g**	**Fibre g/100 g**	**Protein g/100 g**	**Salt g/100 g**
All ready-sliced meat analogues		40	212 (198–247)	8.3 (4.1–13.6)	1.2 (0.7–1.9)	5.9 (3.4–10.1)	1.3 (0.4–2.4)	3.0 (1.1–5.3)	26.1 (16.5–29.4)	1.8 (1.5–2.2)
Nutrition claim	No	18	231 (206–264)*	12.9 (8.7–15.0)*	1.5 (1.1–1.9)*	5.4 (4.4–7.2)	0.9 (0.5–1.7)	2.5 (1.5–5.3)	27.0 (15.0–28.0)	1.9 (1.7–2.3)*
	Yes	22	205 (71–221)	6.3 (1.0–8.5)	1.0 (0.0–1.6)	6.5 (0.1–13.0)	1.5 (0.0–2.8)	3.5 (0.7–5.3)	26.1 (19.2–30.0)	1.6 (1.5–2.2)
Protein claim	No	21	218 (206–257)	13.0 (8.7–15.0)*	1.3 (1.1–1.9)	5.6 (4.4–7.2)	0.9 (0.5–1.4)	2.5 (1.5–5.3)	21.4 (14.9–27.2)*	1.8 (1.7–2.3)
	Yes	19	210 (71–224)	5.8 (1.0–8.1)	0.9 (0.0–1.8)	6.4 (0.1–13.0)	1.7 (0.0–3.2)	3.4 (0.7–5.3)	26.1 (26.1–30.0)	1.5 (1.5–2.2)
Fibre claim	No	33	212 (196–249)	8.1 (4.1–13.8)	1.2 (0.7–1.9)	5.1 (2.8–8.1)	0.9 (0.4–1.7)	2.0 (0.7–4.8)	26.1 (17.5–29.0)	1.8 (1.5–2.2)
	Yes	7	212 (208–221)	12.0 (5.8–13.4)	1.1 (1.0–1.8)	7.0 (6.4–10.9)	2.2 (1.1–3.2)	4.3 (3.5–5.5)	19.2 (6.4–31.0)	2.0 (1.5–2.5)
Organic	No	4	204 (135–249)	10.1 (4.4–16.7)	1.0 (0.5–1.5)	6.0 (2.8–11.2)	1.4 (0.7–1.8)	3.0 (1.6–4.8)	16.5 (11.3–20.5)	1.7 (0.9–2.1)
	Yes	36	214 (199–247)	8.2 (4.1–13.6)	1.2 (0.7–1.9)	5.9 (3.4–10.1)	1.1 (0.4–2.7)	3.0 (1.1–5.3)	27.0 (18.3–29.9)	1.8 (1.5–2.2)

*Data are expressed as median (25°–75° percentile). Asterisks within the same column indicate significant differences between products with or without nutrition claims and organic declaration (Mann–Whitney non-parametric test for two independent samples, p < 0.05)*.

Overall, 59.3% of the PBMA products had pulses as the main ingredient, while 22.7 and 18.0% were mainly based on vegetables (i.e., spinach, carrots, broccoli) and (pseudo)cereals (i.e., wheat, bulgur, quinoa), respectively. Among the PBSMA, 45.0% were based on cereals (i.e., wheat), 25.0% on pulses (i.e., soy), 7.5% on oils (i.e., sunflower oil), and the remaining 22.5% on other ingredients (water emulsions of different ingredients) (data not shown).

As for controls, 25 out of the 225 items retrieved from the online stores were removed because not respect inclusion criteria. Besides these 200 commercial control products, 69 controls were obtained from the Food Composition Database for Epidemiological Studies in Italy (12). Controls were subcategorized in burgers (38.4%), cutlets (26.0%), cured meats (19.3%), meatballs (10.0%), and steaks (6.3%).

### Nutritional Composition of Meat Analogues

Table 1 reports the nutrition information, according to the Council Regulation (EU) n. 1169/2011 (14), for PBMA. The nutrition declaration is reported for each category and product with and without nutrition claims, organic declaration, and gluten-free indication. For nutrition claims, only the most frequent claims are shown (i.e., fat, protein, and fibre claim).

The median energy content of all plant-based meat analogues was 198 (155–230) kcal/100 g, with steaks being the analogues with the lowest content (*p* < 0.05). The median protein content was 14.0 (9.8–17.0) g/100 g, and the steaks had a significantly higher protein content than the other analogues. Overall, analogue steaks were also significantly lower in total fat, saturates, total carbohydrates, sugars, fibre, and salt than the other categories. The other categories (burgers, meatballs, and cutlets) did not differ from each other for total fat, saturates, sugars, fibre, and protein contents (*p* > 0.05). Conversely, they differed from each other for salt, of which cutlets had the highest content (*p* < 0.05). In addition, cutlets also differ from burgers and meatballs for the total carbohydrates, having a significantly higher content.

Products with at least a nutrition claim were significantly lower in saturates and higher in protein than products without a nutrition claim. In particular, as expected, products with a fat claim were significantly lower in total fat and saturates. However, they were also significantly higher in sugars and protein than products without this claim. Similarly, besides having an expected significantly higher protein content, products with a protein claim had significantly lower saturates than their counterparts without this claim. Products with a fibre claim were significantly higher not only in fibre but also in energy, total carbohydrates, sugars, and salt than their counterparts without the claim. Analogues reporting an organic declaration had significantly lower energy, total fat, sugars, fibre, and salt content than conventional items. Products with gluten-free indication were significantly lower in energy, saturates, and salt than analogues without such an indication.

The nutrition information, according to the Council Regulation (EU) 1169/2011 (14), and the nutrition claim and organic declaration for PBSMA are reported in Table 2. For the nutrition claims, only the most frequent claims are shown (i.e., protein and fibre claim).

The median energy content of all these products was 212 (198–247) kcal/100 g, with protein and fats as the main nutrients contributing to the energy content. Products bearing at least a nutrition claim were significantly lower in energy, total fat, saturates, and salt than products without a nutrition claim. The products that reported a protein claim, in addition to being, as expected, higher in protein, were significantly lower in total fat. Intriguingly, products with and without a claim on fibre did not significantly differ for their fibre content. Nutrition declaration of organic products was not different from conventional counterparts.

### Comparison of the Nutrition Facts of Plant-Based Meat Analogues With Meat Counterparts

Table 3 shows the nutrition information of meat controls. Among meats, the highest energy content was found in cutlets, due to the higher total carbohydrate content than the other meat types. Cured meats showed high energy content due to high amounts of total fats and saturates.

**Table 3:** The number of items and nutrition declaration of meat products.

		**Number of items**	**Energy kcal/100 g**	**Total fat g/100 g**	**Saturates g/100 g**	**Total carbohydrates g/100 g**	**Sugars g/100 g**	**Fibre g/100 g**	**Protein (g/100 g)**	**Salt (g/100 g)**
Steaks	Red meats	12	137 (117–145)^c^	5.7 (3.5–7.0)^b^	1.9 (1.2–2.4)^b^	0.0 (0.0–0.0)^c^	0.0 (0.0–0.0)^b^	0.0 (0.0–0.0)^c^	21.1 (20.7–21.3)^a^	0.6 (0.6–0.7)^c^
	White meats	5	120 (107–121)^c^	1.9 (1.2–5.1)^b^	0.6 (0.4–1.9)^b^	0.0 (0.0–0.0)^c^	0.0 (0.0–0.0)^b^	0.0 (0.0–0.0)^c^	23.3 (18.7–24.0)^a^	0.6 (0.6–0.7)^c^
Burgers		103	180 (147–223)^b^	11.0 (7.4–16.0)^a^	4.5 (2.6–6.6)^a^	1.4 (0.5–3.1)^bc^	0.3 (0.0–0.5)^b^	0.0 (0.0–0.5)^bc^	17.0 (16.0–18.2)^b^	1.2 (1.0–1.4)^b^
Meatballs		27	158 (144–204)^b^	9.2 (6.3–14.0)^a^	3.5 (2.3–5.5)^a^	4.0 (2.5–5.3)^b^	0.6 (0.5–1.2)^a^	0.1 (0.0–0.5)^ab^	16.4 (16.0–18.0)^b^	1.5 (1.2–1.6)^a^
Cutlets		70	226 (208–249)^a^	11.0 (9.7–13.6)^a^	1.7 (1.3–2.5)^b^	17.0 (15.0–20.0)^a^	0.8 (0.5–1.2)^a^	0.5 (0.0–1.5)^a^	13.5 (12.0–15.0)^c^	1.4 (1.3–1.5)^a^
Cured meats		52	318 (264–393)	26.0 (19.2–34.0)	8.3 (5.8–10.8)	0.0 (0.0–1.0)	0.0 (0.0–0.9)	0.0 (0.0–0.0)	23.1 (19.6–27.0)	3.8 (2.3–4.8)

*Data are expressed as median (25°–75° percentile). Different letters in the same columns refer to significant differences among categories of meat controls (Kruskal–Wallis non-parametric one-way ANOVA for independent samples with multiple pairwise comparisons test, p < 0.05). Cured meats are not included in the statistical analysis because they are the sole control for plant-based sliced meat analogues*.

**Figure 1:**
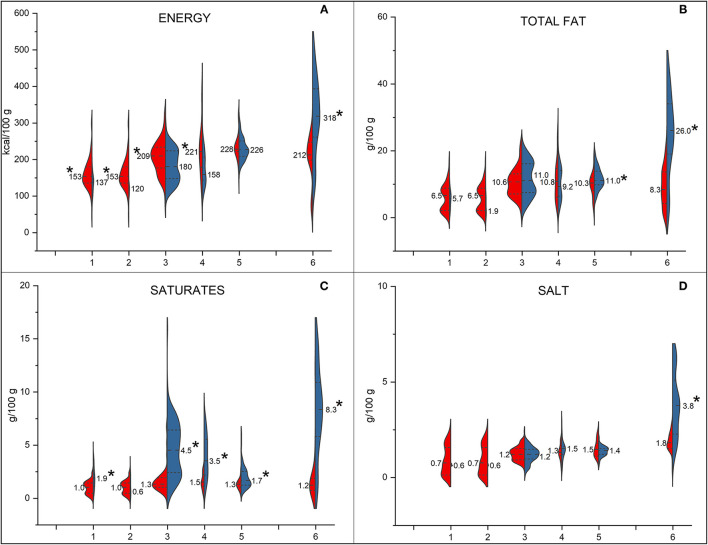
Comparison of nutritional composition—energy **(A)**, total fat **(B)**, saturates **(C)**, and salt **(D)**—between meat analogue products (in red) and their controls (in blue). In each plot are reported the data of steaks, red meat (1) and white meat (2), burgers (3), meatballs (4), cutlets (5), and cured meats (6). For steaks, 0.5 g of added salt per 100 g of product was considered. The asterisk refers to differences between commercial products of the same category, for which the Mann–Whitney non-parametric test for two independent samples was used (*p* < 0.05).

**Figure 2:**
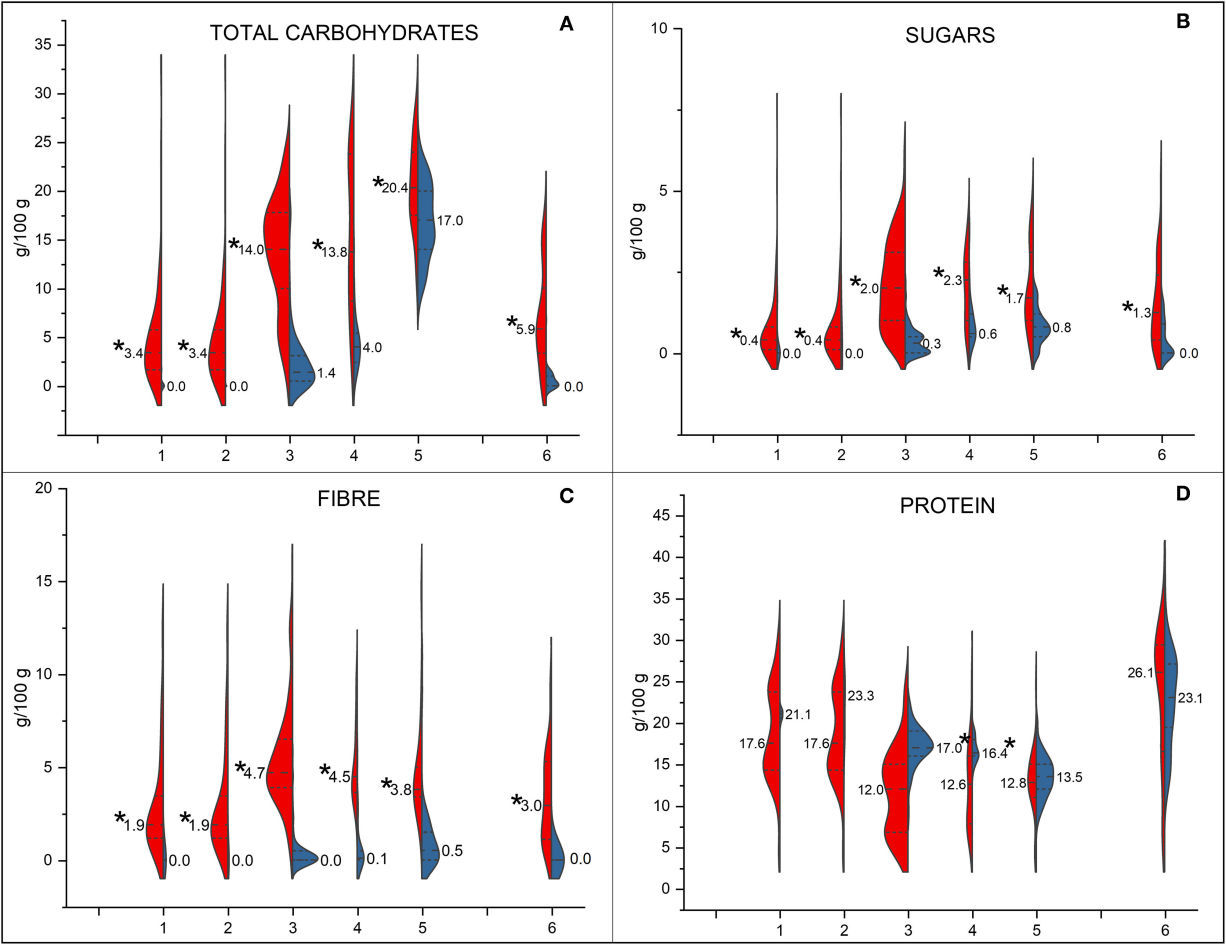
Comparison of nutritional composition—total carbohydrates **(A)**, sugars **(B)**, fibre **(C)**, and protein **(D)**—between meat analogue products (in red) and their controls (in blue). In each plot are reported the data of steaks, red meat (1) and white meat (2), burgers (3), meatballs (4), cutlets (5), and cured meats (6). The asterisk refers to differences between commercial products of the same category, for which the Mann–Whitney non-parametric test for two independent samples was used (*p* < 0.05).

The comparison of nutrition facts of plant-based meat analogues and meat counterparts is graphically shown using split violin plots (Figures 1, 2). Plant-based steaks, burgers, and meatballs showed a higher energy content than controls (*p* < 0.05), while plant-based cured meats showed a significantly lower energy content than controls (Figure 1). Only cutlets and cured meats had a significantly higher total fat content than meat analogues (Figure 1). This difference was not only significant but also very high in the case of cured meats (8.3 vs. 26.0 g/100 g for PBSMA and cured meats, respectively). Meat controls of all the other categories, except for white meat, had a higher saturates content than their analogues (Figure 1).

On the contrary, all the meat analogues showed a higher total carbohydrates, sugars, and fibre content than controls (*p* < 0.05; Figure 2). Among plant-based meats, only burgers and meatballs analogues showed a lower protein content than meat products (*p* < 0.05). The salt content differed only between PBSMA and cured meats (Figure 1). Indeed, PBSMA showed a two-fold lower salt content than cured meats controls (*p* < 0.05).

The analogues showed a median value of 4 ingredients, while the controls did not have any additional ingredients since they are not composite foods. The plant-based burgers had a median number of ingredients of 16 (13–18), higher in the absolute value than the median number [9 (6–10)] found in the commercial meat products. Instead, the plant-based meatballs showed a similar number of ingredients [13 (11–15)] as controls [13 (12–15)] as well as the plant-based cutlets had a similar number of ingredients [14 (12–17)] as meat cutlets [13 (11–15)]. The PBSMA showed a number of ingredients around 13 (8–15). It was not possible to estimate the median number of ingredients for cured meats controls since this information is not provided in the database (12).

### Comparison of the Nutri-Score of Plant-Based Meat Analogues With Meat Counterparts

**Figure 3:**
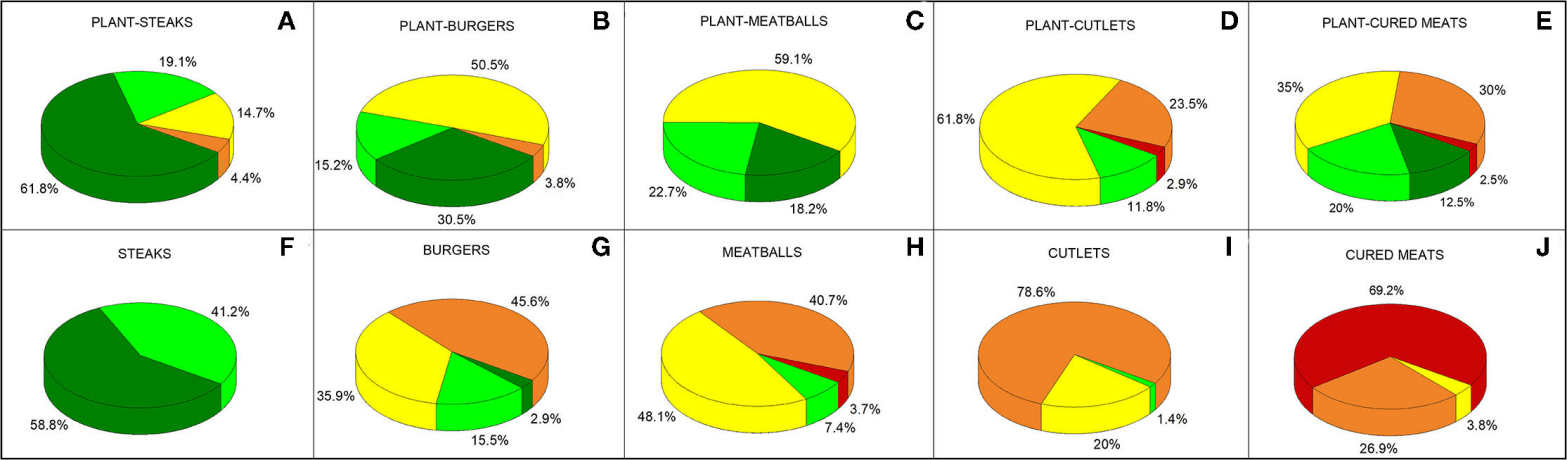
Nutri-score of plant-based analogues **(A–E)** and meat controls **(F–J)**. Data are reported as the percentage of products that have a certain score.

Figure 3 shows the Nutri-Score values of both plant-based analogues and meat products. As for steaks, different added amounts of salt were used, ranging from 0.05 to 0.5 g. As expected, the increased salt content worsened the score, even if the product with the greatest quantity of added salt did not have a score worse than B (data not shown). Compared with plant-based steaks, the score of steak controls (Figure 3F) was reported using the major salt content (i.e., 0.5 g).

Almost all plant-based analogues showed a higher percentage of products having an A and B score than the related meat counterparts. The only exception was found for steaks, with meat products having a score between A and B, while, in plant-based steaks, 19.1% of products had a Nutri-Score C or D. Half of plant-based burgers had a C score and 30.5% of them had an A score. Instead, 46% of meat burgers had a D score and only 2.9% of products had an A score. The majority of plant-based meatballs showed a C score, and the remaining products had A and B scores. Conversely, the meatballs had no products with an A score, only 7.4% had a B score, and almost all products had lower scores than the analogues. The plant-based cutlets had 11.8% of products with a B score and most other products had a C score against the controls, for which the majority of products (78.6%) had a D score. The plant-based cured meats had 32.5% of products with an A and B score, 65% of products with a C and D score. Only 2.5% of plant-based cured meats had an E score, while 69.2% of cured meats had that score. The remaining 30.8% of cured meats had a C and D score.

Data shown in Supplementary Table 2 are relative to the single components used for calculating the Nutri-Score, among which percentages of fruit, vegetables, nuts, and oils are not described in Section Comparison of the Nutrition Facts of Plant-Based Meat Analogues With Meat Counterparts. As expected, this percentage is higher in plant-based meat analogues compared to meat counterparts, in which all the types, except for cutlets, contain a negligible amount. The median Nutri-Score differed in all plant-based meat analogues and cured meats, except for steaks, compared to meat counterparts. In plant-based meat analogues, the better Nutri-Score was partially due to the higher fibre and lower saturates contents compared to meat counterparts. Conversely, the better Nutri-Score in plant-based cured meats was mainly due to the lower saturates and sodium content compared to animal cured meats.

## Discussion

To the best of our knowledge, this is the first survey to evaluate the nutritional quality of several different types of PBMA and PBSMA sold on the Italian market. The critical evaluation of such products falls into a field in great expansion—the conception and development of meat analogue products—seen the consistent promotion of a transition towards plant-based and sustainable dietary models (18). Indeed, the growing attention of the consumer towards these issues has led to the formulation of many new plant-based products that mimic the sensory characteristics of animal-based foods, such as plant-based drinks and meat analogues (6, 19). The main aspects driving consumers' choice towards vegetable alternatives instead of meat products are ethical reasons, public health questions, and environmental issues (20). Concerning the latter, it has been established that a reduction of the consumption of animal-based products, mainly red meat and processed meat, and a switch to plant-based diets may lead to a reduction of mortality by 6–10% and greenhouse emissions by 29–70% in 2050 (21). However, in this frame, data from literature show no substantial differences in terms of greenhouse gas emissions of meat analogues, regardless of their vegetable sources (22, 23).

The market penetration of these products demonstrates the increased interest in PBMA and PBSMA. In the present study, the sole number of retrieved products on the Italian market confirms this growing interest (i.e., 229 PBMA and 40 PBSMA items). The comparison with worldwide data is not easy. Despite the consumer attraction for these products, there are not so many published surveys on the presence of PBMA and PBSMA in the large-scale distribution. A very recent cross-sectional study considered the nutritional facts of 207 meat analogues (among which burgers and meatballs) and 226 meat products available from 14 retailers in the UK (24). Although data were provided by only referring to the specific types of products, the whole energy and macronutrients amounts are pretty in line with the ones found in the present survey. This highlights a significantly lower energy density, total fat, saturated fat, protein, and significantly higher fibre content in PBMA compared to meat products. All these results confirm those from a previous audit survey on the Australian market that considered only vegetable burgers (*N* = 50), which were lower in protein and higher in carbohydrate than meat burgers (25), differently from the findings of the present survey.

Another recent report by “*Safe*food” has identified on the Irish market 354 analogue products, including also other categories not considered in the present survey, i.e., fish substitutes and pastry-based meat substitutes (26). Concerning the nutrition profile, on the whole, the products sold on the Irish market have a similar energy, protein, carbohydrate, total fat, and salt content compared to the Italian ones. On the contrary, Irish plant-based analogues had an almost double average sugars and saturates content than products sold in Italy (26). A Spanish research consulted the “Mintel Global New Product Database” for the 2020 global market of plant-based analogues and pointed out that, in the 86 considered counties, 184 new vegetal burgers have been launched (27). Median values of energy, macronutrients, fibre, and salt are pretty in line with the ones of the present study, except for sugars, which showed a double median value than the one retrieved in the present survey. Higher variability of the observed data has been mostly found for energy, saturates, and carbohydrates values. Authors discussed these variabilities and, in turn, the energy content by considering the presence of various starch-rich ingredients (i.e., flours, starches, and bread-crumbs) as well as oils with different content in saturated fats (27).

Our survey points out that plant-based steaks had the most favourable nutritional characteristics. These steaks had the lowest energy, total and saturated fats, total carbohydrates and sugars, and salt values and the highest protein content, but the lowest fibre content, compared to the other plant-based analogues categories. Except for this last aspect, data from the “*Safe*food” report confirm these better nutritional characteristics of steaks. In that study, these products were grouped together with other types such as mince, meatballs, and Bolognese (26). These better nutritional characteristics of plant-based steaks than other plant-based analogues categories might be explained taking into account the food technology applied for the steak production: the formation during the extrusion process of long fibres and layered structure in steaks, contrariwise to burgers or meatballs, has the advantage of a more organised structure, which does not require the addition of binders and other texturizing agents (28). This might be on the basis of the finding that vegetable steaks have a lower number of ingredients compared to other types of PBMA and, from a nutritional point of view, have a significantly higher protein amount and less total fat and carbohydrates than other PBMA.

Great attention has been given to the protein content of vegetable alternatives by considering that more than 70% of the PBMA and almost the totality of PBSMA boasted a nutrition claim related to the protein content. These values are higher than those found by an Australian survey of analogue products on the US market. Here only 49% of the products retrieved in supermarkets and 40% in online stores boasted a nutrition claim, mainly on the protein content (29). The “*Safe*food” report showed that the protein-related claim in the plant-based analogues sold in Ireland was present in 50% of the products (11% “source of protein” and 39% “high in protein”) (26), while in the paper of Curtain and Grafenauer (25) this value accounted for 60% of the items. The same authors also found a 19 and 21% rate of products with vitamin- and mineral-related claims, respectively. This finding is pretty higher than the one found in the present survey (<4% of the total) and in the US market-based survey by Lacy-Nichols and collaborators, showing <1% of those claims (29). These last data are as interesting as weird by considering that the presence of anti-nutritional factors potentially affects mineral bioavailability. At the same time, as these products are usually fortified with vitamins and, in particular, with vitamin B12 (30), the absence of nutrition claims regarding micronutrient contents was unexpected.

Regarding proteins, by retrieving information from the food pack, we could only collect the protein content and not their quality and source. Soy is generally one of the most common protein sources in PBMA, usually in isolated or concentrated forms, because of different aspects: (i) availability of essential amino acids compared with unprocessed or minimally processed soy protein; (ii) protein digestibility-corrected amino acid score (PDCAAS) close to 1.00, referring value for animal-based proteins; (iii) improvement in colour, flavour and texture parameters of the products (31). Cereals (i.e., rice, barley), pseudocereals (i.e., buckwheat, quinoa), and pulses (i.e., pea, chickpea) are also used as proteins sources mainly because of their textural advantages. However, the vegetable proteins cannot be considered *tout court* animal-derived protein substitutes due to: (i) a different PDCAAS, (ii) presence of trypsin inhibitors—usually inactivated by heat—which affects the protein digestibility, (iii) deficiency in some essential amino acids (32). Besides protein quality, vegetable products have other drawbacks, such as anti-nutritional components (i.e., phytates, oxalates, etc.)—which lower micronutrient bioavailability—and allergenic proteins [i.e., soy, nuts, etc.; (23, 31)].

Moreover, unlike meat which is a protein-rich food, cereal-based products have a higher content of carbohydrates and sugars in comparison with their meat counterparts. As for cereal-based analogues, pulses also lead to having carbohydrates and sugars in the final product. Therefore, these substitutes, which aim to replace meat, also add carbohydrates and sugars to the diet. On the contrary, meat products are not contributors to the intake of sugars, already widely introduced in our diet.

In addition to this, carbohydrates are also added to the products because of their binding capacity. They are added as starches and flours to improve products' texture and gums to improve stability (31). Adding one depends not only on the role it plays in the formulation but also on the product type. While in burgers, meatballs, and cutlets the binding compounds are more often egg protein, methylcellulose, and gluten, in emulsion-based products (as PBSMA) the most commonly used binders are gums and starches (28).

As for carbohydrates, other ingredients are added to the formulations to contribute to the final product. Salt is added as a seasoning but also for toughening the product. Even if in small quantities, the presence of salt involves a change in the structure of the proteins which allows obtaining the desired structure of the final product (28). Our survey found a median salt content of 1.2 and 1.8 g/100 g in PBMA and PBSMA, respectively, meaning that the consumption of these products may largely contribute to reaching and exceeding the 5 g of salt/day recommended by the World Health Organisation. This aspect has been recently highlighted also in the above-mentioned UK cross-sectional study, where authors found a significantly higher salt content in five out of the six PBMA categories compared with their meat analogues. In addition, only around 25% of the surveyed products with a salt content per 100 g below their respective maximum salt reduction targets (24). These results are partially contrasting with the ones of Curtain and Grafenauer (25) based on the products sold on the Australian market: while the sodium content was significantly higher only for plant-based mince items than meat ones, all the other categories of vegetable products, i.e., burgers and sausages, are not significantly different in the sodium content from the animal counterparts (18). Regardless of the differences in sodium content, it is worth to underline that the authors found that most of the products were higher in sodium content compared to the Healthy Food Partnership reformulation targets (25).

Besides comparing the nutrition facts, PBMA, PBSMA, and meats were also compared by the Nutri-Score. This is one of the most common front-of-package labels adopted in several countries. There is still an intense debate whether it is a good way to explore the nutritional quality of food items (31, 33). Nutri-Score should be used to compare products belonging to the same category. However, consumers may perceive it as a tool to compare also products with related alternatives/substitutes. Thus, we used it to compare the nutritional quality of plant-based alternatives with meats. Results clearly showed a higher number of products in the categories A-C for PBMA and PBSMA than meat ones, resulting in healthier products than the meat counterparts. This result is a consequence of the algorithm behind the Nutri-Score that considers saturates and sodium as “negative” components and, conversely, the presence of vegetables and pulses as well as fibre as “positive” components, which inevitably favours plant-based products. However, what must be kept in mind is that the Nutri-Score is based only on the nutritional composition of foods but, for instance, does not consider the number of ingredients of the products and how much they are processed. In fact, it is noteworthy that most of the PBMA and PBSMA products contain several ingredients in their formulations and are produced using several technological processes. For these reasons, all these products are classified as “ultra-processed” foods belonging to the NOVA 4 group (34), a class of products that is under the magnifying glass for their effects on human health (35, 36). As already noted in the literature, Nutri-Score can allow, within the category of ultra-processed foods, to differentiate the nutritional quality of foods, which is essential in terms of health impact (37). Considering that this front-of-pack label only takes into account the nutritional values, our results suggest that several other aspects (e.g., processing, biological value of proteins, presence of many ingredients) should be taken into account for estimating the whole health quality of food products, since none of these dimensions is exclusive and able to summarise, on its own, the overall health value of foods.

Our study has several strengths and limitations worth to be noted. Among strengths, to the best of our knowledge, this is the first survey investigating the nutritional quality and the Nutri-Score of PBMA and PBSMA sold in Italy, retrieving the large majority of products currently on the market. Moreover, focusing on information reported on the food labelling, we were able to focus not only on nutrition declaration but also on other aspects such as the presence of nutrition and health claims and the presence of gluten-free or organic declarations. However, this point can also be considered as a limitation since we cannot consider data on nutrients that are not mandatory based on the current European legislation (e.g., vitamins and minerals). Moreover, we cannot exclude the presence on the marker of other items sold in other minor retailers or other channels (e.g., discounts).

## Conclusions

The first finding of the present survey on the nutritional profile of PBMA and PBSMA sold on the Italian market relates to the huge number of products present nowadays on the market. Moreover, wide variability in the nutritional values among products was observed. This variability was related to the heterogeneity of these products in terms of types and ingredients used. As for animal meats, plant-based steaks resulted nutritionally different from the other categories, showing a higher protein amount and a lower energy, other macronutrients, and salt content. The Nutri-Score values were more favourable in the meat analogues compared to the animal counterparts. Concerning nutritional and health claims and organic ones, as previously reported in other FLIP study papers, a nutritionally significant advantage of PBMA and PBSMA boasting such claims has not been observed compared to their counterparts.

The similarity of the nutritional values of plant-based alternatives and animal meats retrieved by the nutrition facts, and more for steaks, should not induce the consumer to consider PBMA and PBSMA as animal meat equivalent or alternatives. In the present manuscript, as in a previous FLIP manuscript focusing on plant-based beverages vs. milk, it has been widely discussed that the difference in terms of the biological quality of proteins and the micronutrient amounts and bioavailability does not allow to consider PBMA and PBSMA a *tout court* alternative to animal meat. Thus, further studies focusing on evaluating the biological value of protein from plant-based sources and the bioaccessibility of micronutrients are needed. In addition, it is noteworthy the need to improve the formulation of meat analogues in terms of the number of added ingredients, processing, etc. At last, there is also a need for adequate nutritional education programs in order to increase consumers' knowledge and awareness about the differences between animal- and vegetal-based products. All these steps before and during the shopping time are the most important decisional times for the customers, as they drive their intention-to-buy and, finally, the consumption of products, which will directly impact their health.

## Data Availability Statement

The original contributions presented in the study are included in the article, further inquiries can be directed to the corresponding author.

## Sinu Young Working Group

- Margherita Dall'Asta, Department of Animal Science, Food and Nutrition, Università Cattolica del Sacro Cuore, Piacenza, Italy
- Marika Dello Russo, Institute of Food Sciences, National Research Council, Avellino, Italy
- Daniele Nucci, Veneto Institute of Oncology IOV-IRCCS, Padova, Italy
- Stefania Moccia, Institute of Food Sciences, National Research Council, Avellino, Italy
- Gaetana Paolella, Department of Chemistry and Biology A. Zambelli, University of Salerno, Fisciano, Italy
- Veronica Pignone, Freelance Nutritionist, San Giorgio del Sannio, Italy
- Alice Rosi, Department of Food and Drug, University of Parma, Parma, Italy
- Emilia Ruggiero, Department of Epidemiology and Prevention, IRCCS Neuromed, Pozzilli, Italy
- Carmela Spagnuolo, Institute of Food Sciences, National Research Council, Avellino, Italy
- Giorgia Vici, School of Biosciences and Veterinary Medicine, University of Camerino, Camerino, Italy.

## Author Contributions

SC was involved in the protocol design, data analyses, and in the interpretation of results and drafted the manuscript. DA was involved in the protocol design and in the interpretation of the results and contributed to the drafting of the manuscript. TT was involved in the protocol design and critically reviewed the manuscript. NP participated in the conceptualisation of the study, in the interpretation of the results, and critically reviewed the manuscript. DM was involved in the interpretation of the results, conceived and designed the protocol of the study, critically reviewed the manuscript, and had primary responsibility for the final content. All authors contributed to the article and approved the submitted version.

## Conflict of Interest

The authors declare that the research was conducted in the absence of any commercial or financial relationships that could be construed as a potential conflict of interest.

## Publisher's Note

All claims expressed in this article are solely those of the authors and do not necessarily represent those of their affiliated organizations, or those of the publisher, the editors and the reviewers. Any product that may be evaluated in this article, or claim that may be made by its manufacturer, is not guaranteed or endorsed by the publisher.
