# Supplementary material for: Nutritional Quality of Meat Analogues: Results From the Food Labelling of Italian Products (FLIP) Project
Source: Front Nutr. 2022 Apr 26;9:852831. doi: 10.3389/fnut.2022.852831 (PMC9090485; doi:10.3389/fnut.2022.852831)
Supplement: Supplementary file 1 [file Table_1.DOCX]

**Supplementary Information**

**Tab. SI 1** Definitions and examples of the categorization of the meat analogues

|  | | | DEFINITION AND EXAMPLES |
| --- | --- | --- | --- |
| SAMPLES | PLANT-BASED MEAT ANALOGUES | | Plant-based substitutes for meat |
|  | PLANT-BASED READY-SLICED MEAT ANALOGUES | | Plant-based substitutes for cured meats |
| CATEGORY | STEAKS | | Plant-based products resembling steaks (e.g. lupine steaks, tofu, seitan, soy strips) |
|  | BURGERS | | Plant-based products resembling burgers (e.g. spinach burgers, chickpea burgers, patties) |
|  | MEATBALLS | | Plant-based products resembling meatballs (e.g. lentil meatballs, soy meatballs, Mopur® meatballs) |
|  | CUTLETS | | Plant-based products resembling cutlets (e.g. soy nuggets, Mopur® nuggets, mushroom cutlets, soy cutlets) |
|  | CURED MEATS | | Plant-based products resembling cured meats (e.g. lupin frankfurters, soy slices, Mopur® slices) |
| NUTRITION CLAIM | | NO | Products not carrying nutrition claim declaration according to the Council Regulation (EC) 1924/2006 |
|  |  | YES | Products carrying at least a nutrition claim declaration according to the Council Regulation (EC) 1924/2006 (e.g. source of protein, rich in fibre, low in saturated fat) |
| HEALTH CLAIM | | NO | Products not carrying health claim declaration according to the Council Regulation (EC) 1924/2006 |
|  |  | YES | Products carrying at least a health claim declaration according to the Council Regulation (EC) 1924/2006 (e.g. reduces cholesterol, helps maintain normal cholesterol levels) |
| ORGANIC | | NO | Products not carrying organic declaration according to the Council Regulation (EC) No 834/2007 |
|  |  | YES | Products carrying organic declaration according to the Council Regulation (EC) No 834/2007 |
| GLUTEN FREE | | NO | Products not carrying gluten free indication |
|  |  | YES | Products carrying gluten free indication (products showing the wording “gluten free” and the crossed grain symbol) |
