# Supplementary material for: Nutritional Quality of Meat Analogues: Results From the Food Labelling of Italian Products (FLIP) Project
Source: Front Nutr. 2022 Apr 26;9:852831. doi: 10.3389/fnut.2022.852831 (PMC9090485; doi:10.3389/fnut.2022.852831)
Supplement: Supplementary file 2 [file Table_2.docx]

**Tab. SI 2**The number of items and the median values of the components of the Nutri-Score in meat products and meat analogues

|  |  | Number of items | Energy  (kJ/100 g) | Saturates (g/100 g) | Sugar  (g/100 g) | Sodium (mg/100 g) | Protein  (g/100 g) | Fibre  (g/100 g) | F, V, L,  N & Oils (%) | Score | Nutri-Score |
| --- | --- | --- | --- | --- | --- | --- | --- | --- | --- | --- | --- |
| STEAKS | Plant based | 68 | 640 (564-747)^a^ | 1.0 (0.5-1.4)^a^ | 3.4 (1.7-5.7)^a^ | 298 (46-610)^b^ | 17.6 (14.3-23.7) | 1.9 (1.2-3.4)^a^ | 25.0 (3.8-52.2)^a^ | -2 (-5-1) | A (A-B) |
|  | Animal (red meats) | 12 | 573 (488-608)^b^ | 1.9 (1.2-2.4)^ab^ | 0.0 (0.0-0.0)^b^ | 1000 (1000-1000)^a^ | 21.1 (20.7-21.3) | 0.0 (0.0-0.0)^b^ | 0.0 (0.0-0.0)^b^ | -1 (-1-1) | A (A-B) |
|  | Animal (white meats) | 5 | 502 (448-506)^b^ | 0.6 (0.4-1.9)^b^ | 0.0 (0.0-0.0)^b^ | 1000 (1000-1000)^a^ | 23.3 (18.7-24.0) | 0.0 (0.0-0.0)^b^ | 0.0 (0.0-0.0)^b^ | -2 (-2-0) | A (A-B) |
| BURGERS | Plant-based | 105 | 874 (736-975)* | 1.3 (1.0-1.9) | 14.0 (10.0-17.8)* | 480 (360-600) | 12.0 (6.8-15.0) | 4.7 (3.9-6.5)* | 41.2 (20.0-55.2)* | 5 (-1-8) | C (A-C) |
|  | Animal | 103 | 774 (621-935) | 4.9 (2.6-6.6)* | 1.4 (0.5-3.1) | 520 (400-592)* | 17.0 (16.0-18.2) | 0.0 (0.0-0.5) | 1.0 (0.0-3.0) | 5 (3-13)* | C (C-D)* |
| MEATBALLS | Plant-based | 22 | 923 (757-1059)* | 1.5 (1.1-2.1) | 13.8 (8.7-23.8)* | 520 (449-630) | 12.6 (7.9-16.0) | 4.5 (3.6-6.5)* | 46.1 (15.0-55.8)* | 6 (1-8) | C (B-C) |
|  | Animal | 27 | 661 (600-854) | 3.5 (2.3-5.5)* | 4.0 (2.5-5.3) | 600 (480-640) | 16.4 (16.0-18.0)* | 0.1 (0.0-0.5) | 3.0 (0.0-5.0) | 8 (3-13)* | C (C-D)* |
| CUTLETS | Plant-based | 34 | 954 (925-1008) | 1.3 (1.0-1.5) | 20.4 (17.6-24.0)* | 582 (442-704) | 12.8 (11.2-15.0) | 3.8 (3.4-5.0)* | 16.7 (10.0-33.6)* | 9 (8-11) | C (C-D) |
|  | Animal | 70 | 950 (904-1062) | 1.7 (1.3-2.5)* | 17.0 (15.0-20.0) | 560 (480-640) | 13.0 (12.0-15.0) | 0.5 (0.0-1.5) | 8.0 (5.0-10.0) | 12 (11-13)* | D (D-D)* |
| CURED MEATS | Plant based | 40 | 887 (827-1030) | 1.2 (0.7-1.8) | 5.9 (3.5-9.6)* | 720 (600-880) | 26.1 (16.8-29.2)* | 3.0 (1.2-5.3)* | 18.5 (8.0-33.5)* | 9 (1-12) | C (B-D) |
|  | Animal | 52 | 1331 (1106-1644)* | 8.3 (5.8-10.8)* | 0.0 (0.0-1.0) | 1501 (916-1928)* | 23.1 (19.6-27.0) | 0.0 (0.0-0.0) | 0.0 (0.0-0.0) | 20 (17-23)* | E (D-E)* |

Data are expressed as median (25°-75° percentile). Legend: F, V, L, N &Oils: percentage of fruit, vegetables, legumes, nuts and oils. Different letters in the same column refer to significant differences among plant-based and animal steaks (Kruskal–Wallis non-parametric one-way ANOVA for independent samples with multiple pairwise comparisons test, p < 0.05). Asterisks within the same column indicate significant differences between plant-based and animal products (Mann–Whitney non-parametric test for two independent samples, p < 0.05).
